# Correlates of appropriate disposal of children’s stools in Malawi: a multilevel analysis

**DOI:** 10.1186/s12889-020-08725-2

**Published:** 2020-05-01

**Authors:** Owen Nkoka

**Affiliations:** 1 Institute for Health Research and Communication (IHRC), P. O Box 1958, Lilongwe, Malawi; 2 School of Public Health, College of Public Health, Taipei Medical University, Taipei, 110 Taiwan

**Keywords:** Safe stool disposal, Multilevel analysis, Malawi

## Abstract

**Background:**

Management of children’s stools is an important aspect of achieving open defecation free communities and reduction of diarrhea. However, information regarding individual- and community- level factors associated with safe child stool disposal in Malawi is limited. The current study aimed to assess the prevalence of safe child stool disposal and the associated individual- and community- level factors in Malawi.

**Methods:**

The cross-sectional study used data from the 2015–16 Malawi Demographic Health Survey in which 6326 children aged under 2 years, nested within 850 communities, were analyzed. Individual- and community- level factors were tested for association with safe child stool disposal practice using multilevel logistic regression models.

**Results:**

Results revealed that 85.6% of the women reported to have safely disposed of their children’s stools. Women from households with improved sanitation had 36.0% greater odds of safely disposing of their children’s stools compared with those from households with unimproved sanitation [(adjusted odds ratio (aOR): 1.36; 95% confidence interval (CI): 1.12–1.65). Further, women from communities with a middle (aOR: 1.62; 95% CI: 1.18–2.21) and high (aOR: 1.45; 95% CI: 1.14–1.84) percentage of educated women were more likely to have their children’s stools safely disposed of than those from communities with a low percentage of educated women. Children’s age, media exposure, and region were significantly associated with safe stool disposal.

**Conclusion:**

Both Individual- and community-level factors were revealed to be important factors for child stool disposal. Public health strategies designed to promote sanitation/safe child stools disposal need to conduct thorough community assessments to identify community-specific needs/barriers. Additionally, public health practitioners should take into consideration the geographical and wealth inequalities when designing programs aimed to improve safe child stood disposal.

## Background

Water, sanitation and hygiene (WASH) is an important public health issue and a key determinant of disease burden among young children [1]. Lack of sanitation contributes to 10.0% of the global burden of disease including diarrhea [2]. Globally, diarrhea is the second leading cause of death in pre-school aged children with 8.0% of all deaths among under-five children attributed to diarrhea [3]. In low and middle income countries (LMICs), 829,000 deaths in 2016 were WASH-related [1]. Therefore, the prevention of WASH-related diseases such as diarrhea, through the promotion of WASH interventions, is imperative [4].

One of the most important WASH components centers around the realization of open defecation-free (ODF) communities. Safe disposal of children’s stools is an important element in the achievement of ODF status within communities [5]. According to the World Health Organization (WHO), safe disposal of children’s stools is when children use the toilet/latrine, or when children’s stools are put/rinsed in the toilet/latrine, or buried [6]. Unsafe disposal of child stools, defined as rinsing/putting in a drain/ditch, or throwing in the garbage, or leaving in the open [6], increases the risk of exposure to fecal pathogens and has been associated with a 23% increased risk of diarrhea diseases in children [7]. An analysis of data from LMICs revealed that over 50% of households with children under the age of 3 years unsafely disposed of their children’s stools [8]. Considering the importance of safe disposal of stools, and the reported high prevalence of unsafe disposal, the sustainable development goal (SDG) number 6 aims at ending open defecation by 2030 [9, 10].

Even though children’s stools are an important source of fecal contamination within the household environment, they are regarded as not harmful in most cases [11]. It has, however, been reported that exposure to children’s stools may be riskier compared with exposure to adults’ [12]. Contamination of the environment with stools is a key contributor of child mortality and morbidity [13] highlighting the significance of safe child stools disposal.

Malawi experiences enormous challenges in sanitation access [14]. For example, in 2010, approximately 21.0% of Malawian households were reported to dispose of children’s stools unsafely, with 1.1 million people reported to practice open defecation (OD) [8, 15]. However, just like in many sub-Saharan African countries, the disposal of children’s stools has received little attention. The link between OD and diarrhea is well known [16, 17]. A recent Malawian study reported that children under the age of 2 years are at risk of fecal-oral contamination through direct and indirect consumption of contaminated soil [18]. Despite the implementation of comprehensive WASH programs aimed at improving ODF (including the implementation of ODF strategy between 2011 and 2015) [19], Malawi reported an increase in diarrhea rates from 17.5% in 2010 to 22.0% in 2015 [20]. The observed increase in diarrhea rates, and the considerable rate of households unsafely disposing of children’s stools, underscores the importance of reinforcing WASH interventions including safe children’s stools disposal in Malawi.

Factors such as area of residence [21], access to improved sanitation [11] and household wealth [11] were associated with safe child stool disposal practices previously. However, there is a lack of information in literature on community characteristics’ influence on safe child stool disposal behaviors. As previously shown [22–25], community dynamics affect individual risk exposure, resource access, and health behaviors. For example, in Indonesia, women from economically advantaged communities were more likely to have institutional deliveries and adequate antenatal care visits compared with those from economically disadvantaged communities [25].

Thus, the 2015–16 Malawi Demographic Health Survey (MDHS) was used to assess both individual- and community-level factors associated with safe child stool disposal.

## Methods

### Study design and setting

This was a cross-sectional study that analyzed stool disposal of children under the age of 2 years from the 2015–16 MDHS which used a two stage cluster sampling design. The 2008 Malawi Housing and Population Census was the sampling frame for the 2015–16 MDHS survey. In the first stage, a total of 850 clusters (173 clusters in urban areas and 677 in rural areas) were selected with probability proportional to size selection. Households within the selected clusters were then listed. The second stage involved selection of households within the clusters using probability systematic selection criteria. All women of reproductive age (15–49 years) from the sampled households were eligible for interview. Of the 25,146 eligible women, 24,562 were successfully interviewed representing a 98% response rate. Face to face interviews were conducted to collect information regarding child health, water, sanitation and hygiene, and sociodemographic factors. Details of 2015–16 MDHS survey methodology are available elsewhere [20]. The current study was restricted to the women with children under 2 years (*n* = 6326). The youngest child per woman was selected for analysis. The frequencies among this sample revealed that each woman included in the analysis had only one child. There was thus no repetition or multiple children from the same woman. The unit of analysis for this study was children younger than 2 years and their mothers (hereinafter referred to as women).

### Study variables

#### Outcome

The outcome measure “safe child stools disposal” was defined as disposing of child stools by putting or rinsing in a toilet or latrine, or burying them, or a situation where the child used a toilet or latrine [6]. Otherwise, stool disposal was regarded as unsafe.

### Independent variables

The independent variables were selected based on previous studies [11, 21, 26]. The description of the individual- and community-level variables are listed in Table 1. In the current study, a community was defined as the cluster (covering an average of 235 households) from the MDHS.

**Table 1:** Measurement of individual- and community-level variables

Individual-level factors	Description
Sex of the child	Male, Female
Age of the child (months)	≤ 5, 6–11, 12–17, 18–23
Maternal age (years)	15–24, 25–34, ≥35
Number of children ever had	1, 2, 3, 4+
Maternal educational level	No formal education, primary, secondary and higher
Wealth	The MDHS uses principal component analysis to score household items to calculate wealth. The scores are categorized into quintiles from poorest to richest. In this study, richest and rich were grouped as “rich” (upper 40%), middle remained the same (middle 20%), and poorest and poor were grouped as “poor” (lower 40%).
Employed	No, yes
Media exposure	No, Yes (Composite variable categorizing those that reported listening to radio, reading newspaper or watching television at least one a week as “yes” otherwise as “no”)
Religion	Catholics, protestants, Muslims and others
Water source	Unimproved, improved (Improved water source included piped water, boreholes or tube-wells, protected dug wells, protected springs, rainwater, and packaged or delivered water)
Sanitation type	Unimproved, improved (improved included flush toilets, piped sewer system, septic tank, flush/pour flush to pit latrine, ventilated improved pit latrine, pit latrine with slab, and composting toilet)
**Community-level factors**	
Residence	Urban, rural
Region	Northern, Central, Southern
Community wealth	Aggregated from individual-level wealth index defined as the proportion of women who were coming from rich households. The resultant score was categorized using tertiles as low, middle, high.
Community education	Aggregated from individual-level maternal educational level defined as the proportion of women who had primary or above education. The resultant score was categorized using tertiles as low, middle, high.

### Statistical analysis

Stata version 15.0 (Stata Corp LP, College Station, TX, USA) was used for analyses. Distribution of participants’ characteristics according to whether they disposed of their children’s stools safely or not were analyzed using Chi-square tests. All analyses took into consideration the sampling weights and the survey design.

A two-level multilevel logistic regression was used to test for the association between individual- and community-level variables and child stool disposal behavior. Four models were fitted; Null model was an empty model with random intercepts and had no predictors and was used to calculate the total variance of child stool disposal across communities. The subsequent models were used to explain the total variance observed in the null model. Model I and II contained a random-intercept fixed-slope with individual- and community- level factors, respectively. Model III contained a random-intercept fixed-slope and controlled for both individual and community-level factors.

The statistical modelling was three-fold. First, fixed effects were measured to establish the association between selected variables and child stool disposal. Second, random effects were calculated to examine the variation in terms of the outcome across communities. Third, model testing was done to test the goodness-of-fit of each model.

Fixed effects: Adjusted odds ratios (aOR) with 95% confidence interval (CI) were used to report the strength of association.

Random effects: Area variance (AV), intraclass correlation coefficient (ICC), proportional change in variance (PVC), and median odds ratio (MOR) [27] were reported for random effects.

Goodness of fit: The fitness of each model was assessed using Akaike Information Criterion (AIC) with a lower value representing a closer model fit. The models were tested for multicollinearity using variance inflation factor (VIF). There were no multicollinearity problems in the models (all variables’ VIF < 10). Significance level was set at *p* < 0.05. In this report, results from model III have been emphasized because this was the better fit model (i.e. lower AIC).

Additionally, the MDHS collected information from all women of reproductive age in the sampled households such that, a single household may have had more than one woman/child. Therefore, a sensitivity analysis by randomly selecting only one child per household was conducted.

### Ethics statement

The survey protocol was reviewed and approved by the National Health Sciences Research Board of Malawi in Lilongwe, Institutional Review Board (IRB) of ICF Macro, and Centers for Disease Control (CDC) in Atlanta. Informed consent was obtained at the beginning of each interview by the DHS surveyors. The author sought and was granted permission to analyze the DHS dataset. Data for DHS are publicly available and can be requested from https://dhsprogram.com/data/.

## Results

### Prevalence of child stool disposal and distribution of study participants’ characteristics

In total, 6326 children nested within 850 communities were analyzed. Table 2 displays the manner in which children’s stools were disposed of. Results revealed that 85.6% of women reported to have safely disposed of their children’s stools. Among those women who left their child’s stools in the open or did not dispose them, most of them (4.3%) had children aged < 6 mo. Similarly, a high proportion of women who had rinsed their children’s stools in the toilet or latrine were those with children aged > 6 mo.

**Table 2:** Prevalence of children’s stool disposal in Malawi stratified by age

Stool disposal manner	Child age category				Total n ^a^ (%) ^b^
	< 6 mo. n ^a^ (%) ^b^	6–11 mo. n ^a^ (%) ^b^	12–17 mo. n ^a^ (%) ^b^	18–23 mo. n ^a^ (%) ^b^	
Used toilet/ latrine	48 (3.0)	40 (2.4)	46 (2.9)	79 (5.3)	213 (3.4)
Put/rinsed in toilet or latrine	1057 (65.9)	1351 (82.2)	1405 (88.1)	1262 (85.1)	5075 (80.2)
Put/rinsed in drain	268 (16.7)	121 (7.4)	62 (3.9)	51 (3.4)	502 (7.9)
Throw in garbage	120 (7.5)	57 (3.5)	31 (1.9)	46 (3.1)	254 (4.0)
Buried	27 (1.6)	31 (1.9)	32 (2.0)	36 (2.4)	126 (2.0)
Left in open or not disposed	69 (4.3)	35 (2.1)	17 (1.1)	9 (0.6)	130 (2.1)
Other	16 (1.0)	8 (0.5)	2 (0.1)	0 (0.0)	26 (0.4)

^a^ Weighted frequency, ^b^ weighted percentage

The results from Chi-square test displaying the distribution of participants’ characteristics, according to whether they disposed their children’s stool safely or not, are listed in Table 3. A high proportion of women whose children were aged 18–23 months (92.9%) disposed of their children’s stools safely. Additionally, among those that safely disposed of their children’s stools, a high proportion had attained secondary and higher education (89.7%). More women from rich households (89.5%) safely disposed of their children’s stools. It was further observed that a high proportion of women who were exposed to the media (87.5%) had safely disposed of their children’s stools. Those from households with improved water sources (86.2%), or improved sanitation (86.6%) safely disposed of their children’s stools. Finally, among those that safely disposed of their children’s stools, most of them came from communities with a high percentage of women from rich households (89.0%), and from communities with a middle (88.8%) and high (87.2%) percentage of educated women.

**Table 3:** Distribution of study characteristics according to whether child’s stools were safely disposed of: results from Chi-square test

Variable	Safe child stool disposal (***n*** = 6326)		***p***-value
	No (***n*** = 913) n ^a^ (%) ^b^	Yes (***n*** = 5413) n ^a^ (%) ^b^	
*Individual-level factors*			
Sex of the child			0.424
Male	447 (14.0)	2739 (86.0)	
Female	466 (14.9)	2674 (85.1)	
Age of the child (months)			**< 0.001**
≤ 5	473 (29.5)	1132 (70.5)	
6–11	221 (13.5)	1422 (86.5)	
12–17	113 (7.1)	1483 (92.9)	
18–23	106 (7.1)	1376 (92.9)	
Maternal age (years)			0.356
15–24	430 (14.9)	2451 (85.1)	
25–34	333 (13.5)	2133 (86.5)	
≥ 35	150 (15.3)	829 (84.7)	
Number of children ever had			0.195
1	231 (13.7)	1456 (86.3)	
2	169 (13.0)	1138 (87.0)	
3	145 (14.5)	854 (85.5)	
4+	368 (15.8)	1965 (84.2)	
Maternal educational level			**< 0.001**
No formal education	132 (17.4)	628 (82.6)	
Primary	645 (15.2)	3603 (84.8)	
Secondary and higher	136 (10.3)	1182 (89.7)	
Wealth			**< 0.001**
Poor	538 (17.6)	2519 (82.4)	
Middle	161 (13.1)	1072 (86.9)	
Rich	214 (10.5)	1822 (89.5)	
Employed			0.259
No	273 (13.4)	1763 (86.6)	
Yes	640 (14.9)	3650 (85.1)	
Media exposure			**0.016**
No	646 (15.4)	3556 (84.6)	
Yes	267 (12.5)	1857 (87.5)	
Religion			0.673
Catholics	183 (15.7)	984 (84.3)	
Protestants	184 (14.1)	119 (85.9)	
Muslims and others	546 (14.2)	3310 (85.8)	
Water source			**0.009**
Unimproved	161 (18.4)	712 (81.6)	
Improved	752 (13.8)	4701 (86.2)	
Sanitation type			**< 0.001**
Unimproved	215 (18.9)	924 (81.1)	
Improved	698 (13.4)	4489 (86.6)	
*Community-level factors*			
Residence			**< 0.001**
Urban	63 (7.4)	796 (92.6)	
Rural	850 (15.5)	4617 (84.5)	
Region			0.298
Northern	124 (17.1)	601 (82.9)	
Central	375 (14.1)	2286 (85.9)	
Southern	414 (14.1)	2526 (85.9)	
Community wealth			**0.004**
Low	349 (16.2)	1802 (83.8)	
Middle	353 (15.6)	1910 (84.4)	
High	211 (11.0)	1701 (89.0)	
Community education			**< 0.001**
Low	415 (18.0)	1883 (82.0)	
Middle	118 (11.2)	938 (88.8)	
High	380 (12.8)	2592 (87.2)	

Bold means *p*-value< 0.05, all *p*-values from Pearson’s chi-square test, ^a^ Weighted frequency, ^b^ weighted percentage

### Correlates of safe child stool disposal

Table 4 displays the adjusted estimates of the selected factors on safe child stool disposal. Results from model III (final model) revealed that women whose children were aged 6–11 mo. (aOR: 3.06; 95% CI: 2.52–3.72), 12–17 mo. (aOR: 6.81; 95% CI: 5.39–8.60), and 18–23 mo. (aOR: 6.58; 95% CI: 5.18–8.35) were more likely to dispose of their children’s stools compared with those whose children were aged < 6 mo. Additionally, compared with women from poor households, those from middle wealth (aOR: 1.25; 95% CI: 1.01–1.55) and rich (aOR: 1.28; 95% CI: 1.01–1.62) households were more likely to safely dispose of their children’s stools. Women who were exposed to the media had increased likelihood (aOR: 1.20; 95% CI: 1.01–1.44) of safely disposing of their children’s stools compared with those not exposed to the media. Further, women from households that had improved sanitation were more likely (aOR: 1.36; 95% CI: 1.12–1.65) to safely dispose of their children’s stools compared with those from households with unimproved sanitation.

**Table 4:** Multilevel logistic analysis of factors associated with safe stool disposal

Variable	Null model	Model I aOR (95% CI)	Model II aOR (95% CI)	Model III aOR (95% CI)
*Individual-level factors*				
Sex of the child				
Male		1.00		1.00
Female		**0.92 (0.79–1.08)**		0.93 (0.79–1.08**)**
Age of the child (months)				
≤ 5		1.00		1.00
6–11		**3.05 (2.51–3.70)**		**3.06 (2.52–3.72)**
12–17		**6.79 (5.37–8.57)**		**6.81 (5.39–8.60)**
18–23		**6.54 (5.15–8.31)**		**6.58 (5.18–8.35)**
Maternal age (years)				
15–24		1.00		1.00
25–34		1.09 (0.84–1.41)		1.07 (0.83–1.38)
≥ 35		0.98 (0.69–1.38)		0.94 (0.67–1.33)
Number of children ever had				
1		1.00		1.00
2		1.19 (0.94–1.08)		1.20 (0.95–1.53)
3		0.87 (0.65–1.16)		0.89 (0.67–1.19)
4+		0.91 (0.65–1.26)		0.94 (0.68–1.31)
Maternal educational level				
No formal education		1.00		1.00
Primary		0.91 (0.71–1.17)		0.83 (0.64–1.08)
Secondary and higher		1.15 (0.83–1.60)		1.02 (0.73–1.62)
Wealth				
Poor		1.00		1.00
Middle		1.24 (0.99–1.53)		**1.25 (1.01–1.55)**
Rich		**1.32 (1.06–1.63)**		**1.28 (1.01–1.62)**
Employed				
No		1.00		1.00
Yes		0.90 (0.76–1.08)		0.89 (0.75–1.06)
Media exposure				
No		1.00		1.00
Yes		**1.21 (1.01–1.45)**		**1.20 (1.01–1.44)**
Religion				
Catholics		1.00		1.00
Protestant		0.96 (0.74–1.24)		0.97 (0.75–1.25)
Muslims and others		0.94 (0.76–1.16)		0.95 (0.77–1.17)
Water source				
Unimproved		1.00		1.00
Improved		**1.29 (1.02–1.63)**		1.23 (0.98–1.55)
Sanitation type				
Unimproved		1.00		1.00
Improved		**1.38 (1.13–1.68)**		**1.36 (1.12–1.65)**
*Community-level factors*				
Residence				
Urban			1.00	1.00
Rural			**0.67 (0.49–0.92)**	0.73 (0.52–1.65)
Region				
Northern			1.00	1.00
Central			**1.68 (1.27–2.23)**	**1.77 (1.31–2.38)**
Southern			**1.42 (1.09–1.84)**	**1.55 (1.18–2.05)**
Community wealth				
Low			1.00	1.00
Middle			1.08 (0.86–1.36)	1.05 (0.82–1.34)
High			1.17 (0.85–1.54)	0.97 (0.71–1.84)
Community women’s education				
Low			1.00	1.00
Middle			**1.63 (1.22–2.19)**	**1.62 (1.18–2.21)**
High			**1.47 (1.18–1.83)**	**1.45 (1.14–1.84)**
Measures of variation				
Area variance (95% CI)	0.66 (0.49–0.90)	0.70 (0.51–0.96)	0.57 (0.41–0.79)	0.63 (0.45–0.88)
ICC (%)	16.8	17.6	14.8	16.1
PCV (%)	Ref.	−6.06	13.6	4.6
MOR	2.18	2.22	2.06	2.14
Model Fit statistic				
AIC	5309.53	4848.50	5278.58	4831.51

Null model contains no explanatory variables; Model I includes individual-level factors only; Model II includes community-level factors only; Model III includes both individual-level and community-level factors

*aOR* adjusted odds ratio*, CI* confidence internal, *ICC* intraclass correlation coefficient, *MOR* median odds ratio, *PVC* proportional change in variance, *AIC* Akaike information criterion

At the community level, the odds of safely disposing child stools were significantly higher among women from the central (aOR: 1.77; 95% CI: 1.31–2.38) and southern (aOR: 1.55; 95% CI: 1.18–2.05) regions compared with those from the northern region. Additionally, women from communities with a middle (aOR: 1.62; 95% CI: 1.18–2.21) and high percentage (aOR: 1.45; 95% CI: 1.14–1.84) of educated women were more likely to safely dispose of their children’s stools compared with those from communities with a low percentage of educated women.

In the null model, significant variation in child stool disposal among women across communities (σ^2^ = 0.66, 95% CI 0.49–0.92) was observed with an ICC of 16.8% justifying the use of multilevel analysis approach (i.e., variation in terms of safe child stool disposal could be attributed to unobserved community characteristics). Even after adjusting for both individual- and community- level factors, the ICC slightly reduced suggesting that 16.1% of community-level variance were unexplained for safe child stool disposal. This signified that the community-level characteristics included in this study could not adequately explain most of the community variance in safe child stool disposal. The final model further revealed the MOR of 2.14 highlighting the effects of community heterogeneity – i.e., if a woman moved to a community with a higher probability of safe child stool disposal, the median increase in the odds of safely disposing of her child’s stools would be 2.14-fold.

The association between safe child stool disposal and diarrhea was also assessed and the results were not statistically significant (aOR: 1.01; 95% CI: 0.85–1.21) (results not shown).

### Sensitivity analysis

Results from the sensitivity analyses are listed in Additional file 1. In total, 214 children (3.4%) came from the households with more than one child. After selecting one child per household through random selection process, a sample of 6219 was analyzed and results were fairly consistent with the results that included the whole sample.

## Discussion

The study aimed to examine the individual- and community-level factors associated with safe child stool disposal behavior among women in Malawi. Results revealed a high prevalence (85.6%) of safe child stool disposal. Significant individual- and community- level factors associated with safe child stool disposal were further observed. For instance, at individual level, increasing child’s age, and access to improved sanitation were associated with increased odds of safe child stool disposal. At community level, women from communities with a high percentage of educated women were more likely to safely dispose of their children’s stools.

There was a high proportion (85.5%) of women safely disposing of their children’s stools suggesting that 14.5% of women were unsafely disposing of their children’s stools. This represents a drop from 21.0% rate of unsafe child stool disposal reported in 2010 suggesting that Malawi has made tremendous strides on issues related to child stool disposal. This could be attributed to continued efforts in raising awareness, and ODF campaigns that have been comprehensively championed between 2010 and 2015 under WASH programs in Malawi. The 85.6% proportion of women safely disposing of their children’s stools reported in this study is higher compared to 36.9% reported in Ethiopia within the same time period (i.e., 2016) [11]. However, it is important that efforts encouraging safe child stool disposal be strengthened to maintain such high proportions. Identifying factors associated with safe child stool disposal is thus necessary to enable the designing of improved, more efficient, and sustainable programs.

Consistent with studies in Ethiopia [11] and Ghana [21], women with older children were more likely to safely dispose of their children’s stools compared with those with younger children. Children’s behavior and capability tend to change as they get older. Younger children are more likely to be using cloths/nappies and mostly rely on their parents to clean them up. In some cases, this may lead to delayed removal of stools from the household environment. Conversely, as children get older they may express that they need to defecate and therefore get the help of an adult to either use the latrine or defecate in the open where it may be more likely to be removed quickly. Other researchers have also revealed that in many LMICs, people have the myth that a young child’s stools are not particularly harmful [11] thus this may partially help to explain why safe child stool disposal increased with age. Nevertheless, this underscores the need for health workers in WASH programs to emphasize proper disposal of children’s stools among women with younger children in Malawi.

Women from middle and rich wealth households were more likely to safely dispose of their children’s stools compared with those from poor households. This is consistent to studies in India [26, 28]. This may be explained in two parts. First, women from rich households may have resources to afford improved sanitation facilities and having access to improved sanitation has been associated with safe child stools disposal [21]. Second, women from rich households may have access to information [29] through different media sources and thus may be knowledgeable on the importance of safe child stools disposal practices. The current results revealed that women who were from household with improved sanitation and those that had media exposure were more likely to safely dispose their children’s stools. Therefore, efforts to increase access to improved sanitation facilities in Malawi should be encouraged. Additionally, the use of mass media to convey information on safe child stool disposal, and sanitation in general, should be strengthened in Malawi.

At the community level, women from the central and southern regions were more likely to have their children’s stools safely disposed. This underscores the need for WASH programs to strengthen their efforts in the northern region of Malawi. Women from communities with a middle or high percentage of educated women were more likely to safely dispose of their children’s stools. Education has been reported to be a key determinant of health and health behaviors [30, 31]. Women with formal education are more likely to be knowledgeable on different health aspects including safe child stool disposal and a high percentage of these women in a community would result in social influences pertaining health behaviors to those women living within the same environment.

Concerning the ICCs in the current study, 16.8% of the total variance safe child stools disposal could be attributed to community-level factors. Additionally, the ICC in the final model (16.1%) suggested that residual community influences were persistent even after adjusting for individual- and community- level factors. This implies that there are other unmeasured community factors such as community mobilization efforts [32], social capital and networks [33, 34], and cultural and social norms [35] that were not included in this study which may have helped to explain the variation in terms of safe child stools disposal across communities. However, the fairly large ICC values observed in this study are indicative of significant group-level variance as it has been previously suggested that an ICC of ≥2.0% is adequate to warrant multilevel analysis [36].

### Implication of study findings

Findings from this study have some implications for public health practice. First, the current analysis revealed there were unobserved community factors that may influence child stool disposal. Community assessments to identify inherent community factors influencing people’s child stool disposal behaviors should be considered when designing WASH programs in order to come up with context specific programs. Engaging community leaders and local health workers to identify gaps or misconceptions that need to be addressed in order to improve behavior change may be important. Further, innovative methods that may influence behavior change are needed. Second, individual-level factors such as age of the child and media exposure were associated with women’s safe child stool disposal. Thus, programs should emphasize health messages among women with younger children. Additionally, WASH programs should consider strengthening the use of mass media to convey their messages to the populace. Third, improvements of other elements of WASH especially access to improved sanitation is paramount in the process of improving safe child stools disposal. Fourth, the reinforcement of WASH programs that focus on socioeconomically disadvantaged communities may prove beneficial. The regional variations observed in the current study may also reveal underlying inequalities in the distribution of WASH programs in Malawi. Thus, it is important that the northern region should not be left behind in the enhancement of WASH programs.

### Strengths and limitations

Generalizability of the results to Malawian women is a strength as nationally representative data were used. Additionally, the current analysis attempted to examine the community effects as such, child stool disposal varied across communities highlighting the importance of considering community characteristics in WASH programming. Nevertheless, other factors which are “unobserved” at community level (such as social and cultural norms) were not available in MDHS dataset and therefore, could not be examined. As a cross-sectional study, causality could not be inferred. The measurement of child stool disposal relied on self-reports, which may have led to social desirability bias. As such, social desirability bias might have affected the current study’s results as the good behavior of safe child stools disposal might have been over-reported. However, the MDHS interviewers were well trained to skillfully administer the questionnaire and this may potentially help limit the influence of self-report bias. Nevertheless, results from the current study should be carefully interpreted as residual social desirability bias may still have influenced the study findings.

## Conclusion

A high proportion of women in Malawi reported to have safely disposed of their children’s stools. Both individual- and community- level factors were associated with safe child stool disposal in Malawi. Efforts aiming to help communities achieve ODF status through improving child stool disposal practices should consider the individual- and community- level factors. Notably, the observed regional differences in terms of safe child stool disposal suggest that more efforts should be placed in the northern region of Malawi. Future qualitative studies are needed, especially in the northern region of Malawi, to understand the underlying community influences such as social and cultural norms and their role in influencing safe child stool disposal. WASH programs should conduct a thorough community profiling and identify barriers that may hinder safe child stool disposal practices.

## Supplementary information

**Additional file 1: **Multilevel regression results (excluding multiple participants from the same household i.e., *n* = 6219)

## Data Availability

The study used, with permission, data from the International Classification of Functioning, Disability, and Health (ICF). The data is publicly available upon request from the ICF on (https://dhsprogram.com/data/available-datasets.cfm).

## Abbreviations

LMICs: Low and middle income countries
ODF: Open defecation free
OD: Open defecation
aOR: adjusted odds ratio
CI: Confidence interval
MDHS: Malawi demographic health survey
ICC: Intraclass correlation coefficient
PVC: Proportional change in variance
AIC: Akaike information criterion
MOR: Median odds ratio
