# Supplementary material for: Correlates of appropriate disposal of children’s stools in Malawi: a multilevel analysis
Source: BMC Public Health. 2020 May 1;20:604. doi: 10.1186/s12889-020-08725-2 (PMC7195806; doi:10.1186/s12889-020-08725-2)
Supplement: Supplementary file 1 — Additional file 1: Multilevel regression results (excluding multiple participants from the same household i.e., n = 6219) [file 12889_2020_8725_MOESM1_ESM.docx]

| Table S1 Multilevel logistic analysis of factors associated with safe stool disposal (excluding multiple participants from the same household i.e., n = 6219) | | | | | |
| --- | --- | --- | --- | --- | --- |
| Variable | | Null model | Model I  aOR (95% CI) | Model II  aOR (95% CI) | Model III  aOR (95% CI) |
| *Individual-level factors* | |  |  |  |  |
| Sex of the child | |  |  |  |  |
| Male | |  | 1.00 |  | 1.00 |
| Female | |  | **0.93 (0.80 – 1.09)** |  | 0.94 (0.79 – 1.09**)** |
| Age of the child (months) | |  |  |  |  |
| ≤5 | |  | 1.00 |  | 1.00 |
| 6 – 11 | |  | **3.07 (2.52 – 3.74)** |  | **3.08 (2.53 – 3.75)** |
| 12 – 17 | |  | **6.76 (5.35 – 8.55)** |  | **6.77 (5.36 – 8.56)** |
| 18 - 23 | |  | **6.57 (5.17 – 8.36)** |  | **6.60 (5.19 – 8.39)** |
| Maternal age (years) | |  |  |  |  |
|  | 15 – 24 |  | 1.00 |  | 1.00 |
|  | 25 – 34 |  | 1.09 (0.84 – 1.41) |  | 1.06 (0.82 – 1.38) |
|  | ≥ 35 |  | 0.95 (0.67 – 1.34) |  | 0.92 (0.65 – 1.30) |
| Number of children ever had | |  |  |  |  |
|  | 1 |  | 1.00 |  | 1.00 |
|  | 2 |  | 1.22 (0.96 – 1.57) |  | 1.23 (0.97 – 1.57) |
|  | 3 |  | 0.87 (0.65 – 1.16) |  | 0.90 (0.67 – 1.20) |
|  | 4+ |  | 0.93 (0.67 – 1.29) |  | 0.97 (0.70 – 1.34) |
| Maternal educational level | |  |  |  |  |
|  | No formal education |  | 1.00 |  | 1.00 |
|  | Primary |  | 0.92 (0.72 – 1.19) |  | 0.84 (0.65 – 1.09) |
|  | Secondary and higher |  | 1.17 (0.84 – 1.62) |  | 1.03 (0.73 – 1.45) |
| Wealth | |  |  |  |  |
|  | Poor |  | 1.00 |  | 1.00 |
|  | Middle |  | **1.26 (1.01 – 1.56)** |  | **1.27 (1.02 – 1.58)** |
|  | Rich |  | **1.34 (1.08 – 1.66)** |  | **1.31 (1.03 – 1.66)** |
| Employed | |  |  |  |  |
|  | No |  | 1.00 |  | 1.00 |
|  | Yes |  | 0.89 (0.74 – 1.06) |  | 0.87 (0.73 – 1.04) |
| Media exposure | |  |  |  |  |
|  | No |  | 1.00 |  | 1.00 |
|  | Yes |  | **1.20 (1.01 – 1.45)** |  | **1.19 (1.01 – 1.43)** |
| Religion | |  |  |  |  |
|  | Catholics |  | 1.00 |  | 1.00 |
|  | Protestant |  | 0.97 (0.75 – 1.25) |  | 0.97 (0.75 – 1.26) |
|  | Muslims and others |  | 0.95 (0.77 – 1.18) |  | 0.96 (0.78 – 1.19) |
| Water source | |  |  |  |  |
|  | Unimproved |  | 1.00 |  | 1.00 |
|  | Improved |  | **1.31 (1.04 – 1.65)** |  | 1.25 (0.99 – 1.58) |
| Sanitation type | |  |  |  |  |
|  | Unimproved |  | 1.00 |  | 1.00 |
|  | Improved |  | **1.35 (1.11 – 1.65)** |  | **1.33 (1.09 – 1.62)** |
| *Community-level factors* | |  |  |  |  |
| Residence | |  |  |  |  |
|  | Urban |  |  | 1.00 | 1.00 |
|  | Rural |  |  | **0.67 (0.49 – 0.92)** | 0.74 (0.53 – 1.03) |
| Region | |  |  |  |  |
|  | Northern |  |  | 1.00 | 1.00 |
|  | Central |  |  | **1.70 (1.28 – 2.25)** | **1.79 (1.33 – 2.29)** |
|  | Southern |  |  | **1.43 (1.10 – 1.86)** | **1.56 (1.18 – 1.86)** |
| Community wealth | |  |  |  |  |
|  | Low |  |  | 1.00 | 1.00 |
|  | Middle |  |  | 1.09 (0.87 – 1.37) | 1.05 (0.82 – 1.34) |
|  | High |  |  | 1.18 (0.89 – 1.56) | 0.98 (0.71 – 1.34) |
| Community women’s education | |  |  |  |  |
|  | Low |  |  | 1.00 | 1.00 |
|  | Middle |  |  | **1.69 (1.26 – 2.27)** | **1.67 (1.22 – 2.29)** |
|  | High |  |  | **1.49 (1.20 – 1.85)** | **1.47 (1.15 – 1.86)** |
| Measures of variation | |  |  |  |  |
|  | Area variance (95% CI) | 0.66 (0.49 – 0.90) | 0.76 (0.50 – 0.95) | 0.56 (0.40 – 0.79) | 0.62 (0.44 – 0.87) |
|  | ICC (%) | 16.8 | 17.4 | 14.6 | 15.8 |
|  | PCV (%) | Ref. | -4.5 | 15.2 | 6.06 |
|  | MOR | 2.17 | 2.21 | 2.05 | 2.12 |
| Model Fit statistic | |  |  |  |  |
|  | AIC | 5228.39 | 4771.44 | 5195.16 | 4753.78 |
| Null model contains no explanatory variables; Model I includes individual-level factors only; Model II includes community-level factors only; Model III includes both individual-level and community-level factors  *aOR* adjusted odds ratio*, CI* confidence internal, *ICC* intraclass correlation coefficient, *MOR* median odds ratio, *PVC* proportional change in variance, *AIC* Akaike information criterion | | | | | |
